# Systemic health worker suicide prevention–Reframing mental ‘Illness’ to mental injury

**DOI:** 10.1371/journal.pmen.0000150

**Published:** 2024-10-09

**Authors:** Maria Heliana Ramirez

**Affiliations:** Hostile Workplace Recovery, LLC, Santa Clara, California, United States of America; PLOS: Public Library of Science, UNITED KINGDOM OF GREAT BRITAIN AND NORTHERN IRELAND

In the United States, suicide is the second leading cause of death for medical residents in training, [1] however, registered nurses, healthcare support workers, and mental health technicians have even higher rates than physicians, with especially elevated rates among women [2]. Suicide prevention addressing coping rather than occupational hazards is insufficient [3]. Institutional risk factors include hostile patients, extreme work hours, collegial bullying, sleep deprivation, inadequate resources, licensure reporting requirements, and understaffing. For women health workers of color, racial battle fatigue [4] and sexism also activate suicidal crisis [5].

A Center for Disease Control study comparing health worker experiences between 2018 and 2022 revealed that bullying, harassment, and threats from patients and coworkers doubled, burnout increased from 32% to 46%, and harassed workers experienced 5x more anxiety, 3x more depression, and 6x more burnout than non-harassed workers [6].

State medical board licensing requirements to disclose mental health conditions incorrectly assume incompetence, violate the Americans with Disabilities Act, [7] and reduce help-seeking [8]. Despite the launch of the 988-crisis line in 2022, suicide rates continue to rise in the United States. In 2023 a record 50,000+ suicides were recorded- more deaths than any year since records began in 2001 [9]. Health workers may avoid 988 for licensing and personal safety reasons given the reported four-fold increase of police involvement and involuntary hospitalization by 988 over the 1800-Lifeline [10].

Police involvement may be especially worrying for health professionals of color considering reports of Black people being disproportionately shot by police during mental health crisis calls [11]. Some Latinos may also avoid crisis lines fearing police involvement in Immigration and Customs Enforcement and deportation of undocumented family, neighbors, or DACA health providers (i.e., Deferred Action for Childhood Arrivals) [12]. Additionally, health workers of color may avoid emergency rooms and psychiatric facilities care given disproportionate incidence of coercive physical and medical restraint of patients of color [11]. 988’s funder the Substance Abuse Mental Health Services Administration (SAMHSA), informed Congress that “no research validates 988Lifeline’s emergency intervention policy—while some research suggests psychiatric hospitalization may well heighten suicidality.” [10] Beyond licensing and safety, occupational stigma also prevents help-seeking.

Physicians’ culture of “medical exceptionalism, medicalization, and individual responsibility” promotes burnout [13]. Physicians are, trained to be “self-negating,” are stigmatized for disclosing work-related mental injury, [13] and physicians of color face systemic racism via scrutiny of and surveillance over their mental health [14]. Injury from medical culture can be fatal as exemplified by Dr. Nakita Mortimer, a Haitian-American anesthesiology resident who died by suicide in 2023.

Fighting to improve working conditions, Dr. Mortimer organized a union to establish physician training work-hour limits, livable wages, and employee wellness [15]. Dr. Mortimer suffered mental injury from Academic Hazing, a term describing “practices that encourage…overworking and acceptance of burnout from unreasonable and exploitive expectations” [15]. Observing her passing, the Association of Black Women Physicians opined that:

The culture of medicine must shift. Realistic work hours, demands and responsibilities, access to confidential mental health services, commitment to physician wellness and inclusive, nontoxic work environments all need to become a part of a healthy normalization for our profession.

Dr. Mortimer’s death epitomizes occupational demoralization syndrome acquired from the “diseased system for which we work” [16].

## Micro and macro systemic suicide prevention

Existing suicide prevention encourages 911/988 calls and emergency room visits for high-risk suicidal crisis. This approach lacks a trauma-informed, neuroscientific understanding that the suicide-related “fight, flight, and freeze” nervous system response is often amplified (not reduced) by armed officers and busy ERs with extended wait times. Suicide prevention efforts also ignore occupational hazards with a sole focus on coping skills, which may increase suicidal crisis in lieu of medical culture that causes and then stigmatizes mental injury. Work-activated suicidal thoughts signify an occupational *mental injury—*not illness, a stigma-reducing attribution to injurious work conditions not victim-blaming.

Suicide is often stigmatized as weakness but is rather, an involuntary and unconscious nervous system response to overwhelming danger [17]. Suicidal crisis emerges when the brain’s alarm system (amygdala), becomes activated and the brain’s thinking area (prefrontal cortex), goes offline, rendering cognition-based suicide prevention inaccessible. The Global Medical Response’s “Polyvagal Theory and Emergency Responders: How our Nervous Systems Impact our Lives” explains that,

Emergency responders frequently face critical incidents and crisis situations. Their bodies and minds are trained to react in the moment. However, most are not trained how to wind back down after a call, returning to a baseline of calm and safety. (p. 1–2)

Somatic body-based practices engaging the Vagus nerve from stress hormone flooding to a state of rest and repair [17] is a missing link in most suicide prevention.

## Systemic health worker suicide prevention

Systemic suicide prevention is needed at the micro-employee nervous system and macro-employer healthcare system levels with resources that avoid professional barriers to care, increase a sense of safety in one’s body, and reduce isolation and shame. Unlike existing suicide prevention [18], this approach avoids threats to licensure and cognitive behavioral approaches that require critical thinking skills often inaccessible during acute suicidal crisis. The following is a non-exhaustive list of examples and does not replace medical advice from licensed clinicians.

## Employee micro-level suicide prevention: Vagus emergency breaks for acute crisis

1. **Breathing exercises** can positively impact cognitive processing during acute suicidal crisis. The human brain comprises 2% of body weight yet uses 20% of the body’s oxygen when at rest. Regulated breathing is the quickest way to help the brain function at its highest capacity. Longer exhalation than inhalation shifts the nervous system from emergency mode to safety mode by interrupting the stress hormone flooding brain/body feedback loop.
2. **Cold water exposure** can quickly shift mood [19]. Dr. Ursula Whiteside a psychologist with mental health lived experience, created ‘NowMattersNow’ with demonstrations of cold-water exposure and online self-help suicide prevention courses.
3. **Somatic Awareness** of physical sensations and emotions preceding suicidal thoughts is essential. When bodily sensations of stress, panic, or looping pre-suicidal thoughts of isolation or worthlessness emerge, the brain’s Task Positive Network (TPN) can be activated to release feel good hormones (e.g., dopamine, serotonin, endorphins, and oxytocin) to prevent or dampen suicidal thoughts. TPN activities involve attention and goal-oriented behavior (e.g., playing an instrument, preparing a meal, and singing). Eating cultural foods reminiscent of a time when one felt safer and recalling details of a proudest or happiest moment, releases feel good hormones as if the event were happening now.
4. **Reduce toxic exposure** (e.g., coworkers, family, and social media) and increase **group level nervous system regulation**. The following resources are not therapy, avoid licensing reporting, and do not impact medical or insurance records.
  - **Physicians Anonymous (PA),** is a science-based and confidential “third-party group offering both prevention and management of physician burnout and distress” including suicide prevention through anonymous meetings and coaching. PA was created by a physician who survived workplace bullying and provides continuing medical education certificates for anonymous meeting attendance.
  - **The Safe Place App** provides virtual support for Black people experiencing suicidal crisis. Designed by Jasmin Pierre, a Black woman suicide survivor, the app is available at no cost. In addition to support groups, the app also provides Inspirational Black Quotes, Breathing Techniques, Meditation, Videos about Mental Health, Open Forum Discussions, and Tips to Coping after Police Brutality.
  - **Somatic Abolitionism** sessions, the BlackOctopusSociety online community, and book “My Grandmother’s Hands: Racialized Trauma and the Pathway to Mending Our Hearts and Bodies,” by social worker and trauma therapist, Dr. Resmaa Menakem, are examples of resources that provide nervous system toning exercises for racial trauma.

## Healthcare system suicide prevention

Employers must create non-injurious working conditions with reasonable work hours, reduced administrative burden, affordable healthcare, and “debt relief for crushing student loans” [6]. All states should implement the Federation of State Medical Board’s 2018 recommendation to stop asking about physician’s mental health [6]. Employees need “work-limit protections, [to make] occupational health a top-level priority on par with patient safety, and addressing social determinants of both patient illness and clinician burnout” [15] and anti-stigma to reduce barriers to help seeking [4]. Beyond micro and macro-systemic suicide prevention, research is also needed to learn what has helped healthcare workers survive suicidal thoughts. I am therefore calling for research on suicide prevention at the employee nervous system *and* employer healthcare system levels.
